# Respiratory impact of local anaesthetic volume after interscalene brachial plexus block with extrafascial injection: a randomised controlled double-blinded trial

**DOI:** 10.1016/j.bja.2024.12.010

**Published:** 2025-01-23

**Authors:** Yves Renard, Sina Grape, Erin Gonvers, Jean-Benoit Rossel, Patrick Goetti, Eric Albrecht

**Affiliations:** 1 Department of Anaesthesia, University Hospital of Lausanne and University of Lausanne, Lausanne, Switzerland; 2 Department of Anesthesia, Valais Hospital, Sion, Switzerland; 3 Centre for Primary Care and Public Health (Unisanté), Lausanne, Switzerland; 4 Department of Orthopaedic Surgery, University Hospital of Lausanne and University of Lausanne, Lausanne, Switzerland

**Keywords:** brachial plexus block, diaphragm, postoperative analgesia, regional anaesthesia, pain

## Abstract

**Background:**

We have previously demonstrated that an extrafascial injection of 20 ml of local anaesthetic for interscalene brachial plexus block (ISB) reduces the rate of hemidiaphragmatic paralysis by 70% compared with an intrafascial injection, with similar efficacy. In this double-blind trial, we tested the hypothesis that a local anaesthetic volume of 10 ml injected extrafascially would reduce the rate of hemidiaphragmatic paralysis *vs* a volume of 20 ml, while providing similar analgesia.

**Methods:**

Sixty ASA physical status 1–3 patients scheduled for elective shoulder surgery under general anaesthesia were randomised to receive ultrasound-guided extrafascial ISB using ropivacaine 0.75% 20 ml (control group) or 10 ml (low-volume group) injected lateral to the brachial plexus sheath. The primary outcome was incidence of hemidiaphragmatic paralysis (diaphragmatic excursion reduction of >75%), measured by M-mode ultrasonography, at 30 min after the procedure. Secondary outcomes included duration of analgesia and i.v. morphine consumption at 24 h after surgery.

**Results:**

The 30-min hemidiaphragmatic paralysis rate was 80% (95% confidence interval [CI] 61–91%) in the control group and 19% (95% CI 8–40%) in the low-volume group (*P*<0.001). Participants in the low-volume *vs* control group had a shorter duration of analgesia (550 *vs* 873 min; *P*<0.01) and higher i.v. morphine consumption (20 *vs* 12 mg; *P*=0.03).

**Conclusions:**

A low volume of local anaesthetic injected extrafascially reduced the rate of hemidiaphragmatic paralysis, but at the expense of a shorter duration of analgesia compared with standard-dose extrafascial anaesthetic injection.

**Clinical trial registration:**

NCT04726280.

**Editor's key points**

• The optimal local anaesthetic volume balancing safety and efficacy in extrafascial interscalene brachial plexus block is unclear.
• In this study, reducing the anaesthetic volume from 20 ml to 10 ml reduced the rate of hemidiaphragmatic paralysis from 80% to 19%, although analgesia duration was reduced, and morphine consumption was increased.
• The volume of local anaesthetic used in extrafascial interscalene brachial plexus block can be modified to balance analgesia duration against the risk of diaphragmatic impairment.

We have previously demonstrated that an injection of 20 ml of local anaesthetic administered a few millimetres lateral to the fascia surrounding the brachial plexus in the interscalene region provides satisfactory pain control during and after shoulder surgery.^1^ We have also reported that this extrafascial approach to interscalene brachial plexus block (ISB) offers an analgesic efficacy similar to that of an intrafascial injection while reducing the rate of hemidiaphragmatic paralysis by 70% after 30 min^2^ and by 25% after 24 h.^3^

In this randomised, controlled, double-blinded trial, we tested the hypothesis that a local anaesthetic volume of 10 ml injected extrafascially would further reduce the rate of hemidiaphragmatic paralysis compared with a volume of 20 ml, while providing similar analgesia.

## Methods

### Recruitment and randomisation

This trial was approved by the Ethics Committee of Lausanne University Hospital (Commission cantonale d'éthique de la recherche sur l'être humain, protocol number 2020-02930), prospectively registered on clinicaltrials.gov (NCT04726280), and monitored by an independent person from our University Hospital who was not involved in the study. All patients aged 18–85 yr scheduled to undergo elective shoulder surgery (coracoid bone block transfer, arthroscopic rotator cuff repair, other) at Lausanne University Hospital between April 2021 and September 2023 were eligible to participate. Exclusion criteria included existing neurological deficit in the upper limb, history of neck surgery or radiotherapy, moderate-to-severe pulmonary disease (grade III or IV dyspnoea), chest deformity, contraindications to peripheral nerve block (e.g. allergy to local anaesthetics, coagulopathy, infection in the area), and pregnancy. After providing written informed consent, participating patients were randomly allocated on the day of surgery to either the control group (standard volume, 20 ml) or the experimental group (low volume, 10 ml) using a computer-generated randomisation table in aggregates of 10. Assignments were concealed in a sealed, opaque envelope.

### Interscalene block procedure

**Fig 1:**
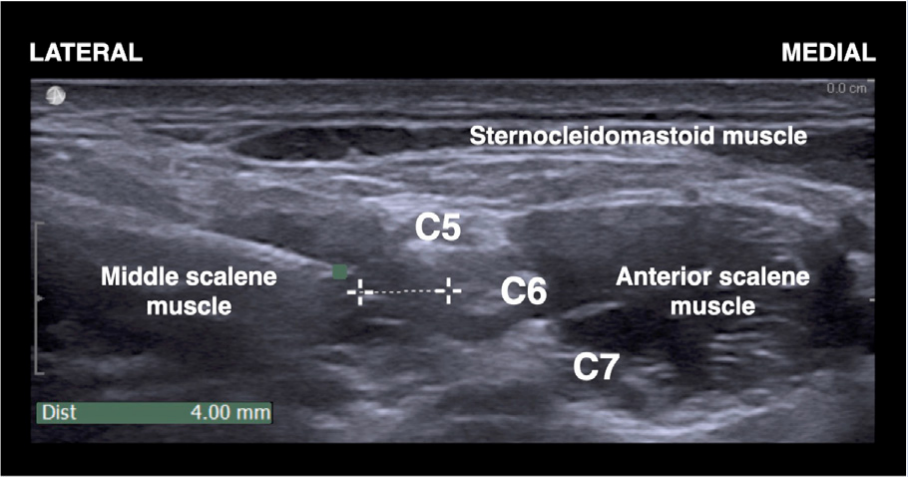
Ultrasound-guided interscalene brachial plexus block with an extrafascial injection: needle tip position at a distance of 4 mm from the lateral border of the brachial plexus sheath; C5, C5 root; C6, C6 root; C7, C7 root.

Ultrasound-guided ISB was performed before surgery in a dedicated block procedure room. All ISBs were achieved or directly supervised by one of the authors (EA), who had no further involvement in the study protocol. Patients were positioned supine with the head turned 45 degrees to the non-operative side. Electrocardiogram, pulse oximetry, and blood pressure monitors were routinely applied, and oxygen was provided. Peripheral i.v. access was established, and midazolam 1–4 mg i.v. was administered for anxiolysis and sedation as needed. The needle insertion site was sterilised with a solution of chlorhexidine 2% in isopropyl alcohol 70%. Under sterile conditions, a high-frequency linear array transducer (18–6 MHz, HF Linear Array 8870; BK Ultrasound, Pea-body, MA, USA) was placed over the interscalene region to visualise the carotid artery and brachial plexus in the short-axis view. After identification of the C5, C6, and C7 roots, a 22-gauge 50-mm insulated block needle (SonoPlex Stim cannula; Pajunk®, Geisingen, Germany) was inserted in-plane on the lateral side of the transducer. The needle was then advanced through the middle scalene muscle and towards the lateral border of the brachial plexus sheath under direct ultrasound guidance. The brachial plexus sheath was identified as the linear hyperechoic layer surrounding the roots of the brachial plexus. The final needle tip position was 2–4 mm lateral to the brachial plexus sheath at a level equidistant between the C5 and C6 roots, measured with the on-screen calliper tool (Fig. 1).^2^ According to group allocation, patients then received either 20 ml or 10 ml of ropivacaine 0.75%, injected in 5-ml increments with intermittent aspiration. No dose adjustments were made based on patient age. We did not intend to avoid the spread of local anaesthetic between the anterior scalene and the sternocleidomastoid muscles, and therefore towards the phrenic nerve. The needle tip was never repositioned, except if patients complained of paraesthesia.

### Intraoperative and postoperative procedure

After application of routine monitors in the operating theatre, patients received a standard general anaesthetic administered by an anaesthesiologist who was blinded to group allocation. Anaesthesia was induced using sufentanil 0.1 μg kg^−1^ i.v. and propofol 2–4 mg kg^−1^ i.v. with tracheal intubation facilitated by rocuronium 0.6 mg kg^−1^ i.v. Maintenance of anaesthesia was via inhaled sevoflurane 1.6–2.4% in a 40:60 mixture of oxygen and air. Positive pressure ventilation was initiated with tidal volume and rate adjusted to maintain an end-tidal carbon dioxide pressure of 35–40 mm Hg. Sufentanil 2.5 μg i.v. was administered as needed to treat increases in blood pressure or heart rate of more than 15% above preinduction baseline values. As per our routine institutional practice, all patients received magnesium sulphate 50 mg kg^−1^ i.v.^4^ and dexamethasone 0.15 mg kg^−1^ i.v.^5^ at the beginning of the surgery, and ketorolac 30 mg i.v., paracetamol 1 g i.v., and ondansetron 4 mg i.v. at the end of surgery for multimodal analgesia and antiemetic prophylaxis.^6^^,^^7^ Muscle relaxation was antagonised with neostigmine 50 μg.kg^−1^ and glycopyrrolate 5–10 μg-kg^−1^ before extubation. In phase 1 recovery, pain (numeric rating scale [NRS] ≥4 or patient request for analgesia) was treated with morphine 1–2 mg every 10 min as needed. Once oral intake was initiated, patients received paracetamol 1000 mg every 6 h and ibuprofen 400 mg every 8 h, along with morphine i.v. patient-controlled analgesia. Antiemetic medications on the ward included ondansetron 4 mg i.v. and metoclopramide 10 mg i.v.

### Block assessment and definition of successful block

Assessment of sensory and motor blocks was performed by a blinded research assistant every 5 min after the injection of local anaesthetics and continued for at least 30 min or until the block was fully effective. Sensory block was tested in the C5 and C6 dermatomes using a blunt tip needle pinprick test (0, no perception; 1, decreased sensation; 2, normal sensation). Motor block was tested using arm abduction (C5) and forearm flexion (C6) (inability to overcome gravity, 0; reduced force compared with contralateral arm, 1; no loss of force, 2). A successful block was defined as complete sensory (score, 0) and motor (score, 0) block in the distribution of the C5 and C6 nerve roots.

### Hemidiaphragmatic excursion and respiratory function assessment

Hemidiaphragmatic excursion was assessed with a low-frequency curvilinear transducer (2–5 MHz, HF Linear Array 8870; BK Ultrasound) using a subcostal approach as described previously.^8^ Briefly, patients were examined in the lying position, and the hemidiaphragm was identified as an hyperechoic line with breathing-related movements using the liver or spleen as an acoustic window. Hemidiaphragmatic excursion was measured by real-time M-mode ultrasonography from the resting expiratory position to a deep and quiet inspiration. Respiratory function was assessed using a bedside spirometer (EasyOne™ Spirometer; ndd Medical Technologies, Andover, UK). After being given standard instructions, the patient was asked to inspire maximally and blow into the device as fast and strongly as possible while sitting in an upright position. The test was repeated three times, and the best value was recorded.

### Outcomes

The primary outcome was the rate of hemidiaphragmatic paralysis 30 min after the procedure. Hemidiaphragmatic paralysis was defined as a >75% reduction in hemidiaphragmatic excursion compared with the pre-procedure value.^9^^,^^10^ Secondary outcomes included respiratory-related, block-related, and pain-related outcomes. Hemidiaphragmatic excursion was also measured before surgery and at 2 and 24 h after surgery. Respiratory-related outcomes included forced vital capacity, forced expiratory volume in 1 second, and peak expiratory flow, all measured 30 min after the injection, in phase 1 recovery at 0–2 h after surgery, and at 24 h after surgery. Block-related outcomes included the following: rates of successful block at 30 min after the injection; rates of paraesthesia during the block procedure; rates of dyspnoea, Claude Bernard-Horner syndrome, and hoarseness at 2 and 24 h after surgery; durations of sensory block (defined as time from the injection of local anaesthetic to the time the patient recovered sensation over the shoulder), motor block (defined as time from injection of local anaesthetic to the time the patient could raise their arm) and analgesia (defined as time from block completion to the time to first dose of i.v. morphine); rest and dynamic pain scores on an NRS (out of 10) at 2 and 24 h after surgery; cumulative postoperative i.v. morphine consumption at 2 and 24 h after surgery; and satisfaction with the overall anaesthetic management (NRS out of 10).

As per our routine institutional practice, all patients were hospitalised overnight and evaluated at 24 h after surgery. Patients were also contacted on postoperative day 7 to capture information on any block-related complications such as haematoma, infection, persistent paraesthesia, or weakness in the upper limb.

The patients, anaesthetists administering the general anaesthetic, phase 1 recovery nurses, ward nurses, and the research assistant measuring the respiratory data and collecting all other data were blinded to the group allocation.

### Sample size calculation

Based on our recently published data, the average rate of hemidiaphragmatic paralysis with an extrafascial injection of 20 ml of local anaesthetics was 21%, with an upper confidence interval (CI) of 46%.^2^ Assuming a 10% reduction rate from the upper CI (46%), an alpha error of 0.05, and a power of 80%, we calculated that 48 participants (24 per group) would be required to detect a between-group difference in the rate of hemidiaphragmatic paralysis. The target sample size was set at 60 participants to allow for a 25% drop-out rate.

### Statistical analysis

Data were analysed on an intention-to-treat basis. Categorical variables are presented as frequencies, and continuous variables are summarised as mean with 95% CI or median with interquartile range (IQR), as appropriate. Distributions of the continuous data (normal or not normal) were determined with a Shapiro–Wilk test. Between-group comparison of continuous data was performed using Student's *t*-test or a Mann–Whitney–Wilcoxon test, depending on their distribution. Categorical and dichotomous data were compared using Fisher's exact test. Survival analysis of the effect of injection volume on time to first opioid request was performed using the Kaplan–Meier method, and the two groups were compared using a log-rank test. Statistical significance was defined as a two-sided *P*-value of <0.05. Statistical analyses were performed using the Stata software (version 16.1; StataCorp, College Station, TX, USA).

## Results

**Fig 2:**
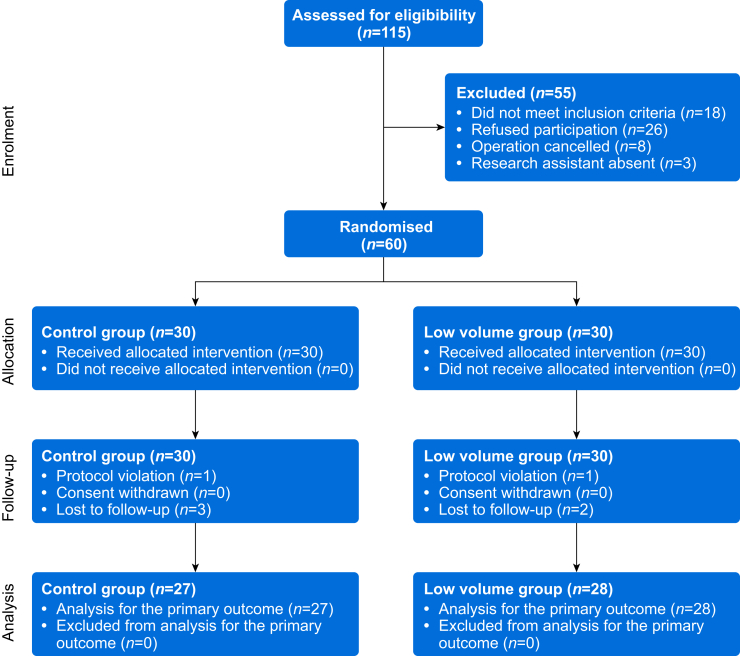
Patient flow chart.

Sixty participants were recruited, and all had an ISB without complaining of paraesthesia during the procedure (Fig. 2). The needle tip was therefore never repositioned. Because of operating theatre time constraints, hemidiaphragmatic excursion was not measured in three participants in the control group and two in the low-volume group. Therefore, 55 patients completed the study for the primary outcome (14.5% female, age 33–49 years) (Table 1). All of these 55 participants had a successful block 30 min after the injection.

**Table 1:** Participant characteristics and clinical data at baseline. Values are mean (95% confidence interval) or number of patients (%). ASA, American Society of Anesthesiologists.

	Control group (*n*=27)	Low-volume group (*n*=28)
Female sex, *n* (%)	5 (19)	3 (11)
Age, yr	43 (36–49)	40 (33–47)
Weight, kg	76 (72–81)	81 (75–86)
Height, cm	174 (171–177)	176 (173–180)
Body mass index, kg m^−2^	25 (24–27)	26 (24–28)
ASA physical status, *n* (%)		
1	8 (30)	9 (32)
2	18 (66)	19 (68)
3	1 (4)	0 (0)
Duration of surgery, min	96 (78–114)	89 (74–103)
Surgical procedure, *n* (%)		
Coracoid bone block transfer (Latarjet procedure)	13 (48)	15 (54)
Arthroscopic rotator cuff repair	9 (33)	10 (36)
Other	5 (19)	3 (11)

The rate of hemidiaphragmatic paralysis at 30 min was 80% (95% CI 61–91%) in the control group and 19% (95% CI 8–40%) in the low-volume group (*P*<0.001). At 2 h after surgery, hemidiaphragmatic paralysis rates in the control and low-volume groups were 67% (95% CI 48–81%) and 21% (95% CI 10–40%), respectively (*P*=0.001). All participants with hemidiaphragmatic paralysis had fully recovered at 24 h after surgery. There were no differences in respiratory-related outcomes during the study (Table 2).

**Table 2:** Respiratory-related outcomes. Values are mean (95% confidence interval) or number of patients (%).

	Control group (*n*=27)	Low-volume group (*n*=28)	*P*-value
**Pre-procedure (baseline)**			
Forced vital capacity, L	4.1 (3.8–4.5)	4.6 (4.2–5.0)	0.06
Forced expiratory volume in 1 s, L	3.3 (3.0–3.6)	3.6 (3.3–3.9)	0.19
Peak expiratory flow, L s^−1^	7.6 (6.8–8.4)	8.1 (7.2–9.0)	0.43
**30 min after block**			
Forced vital capacity, L	3.0 (2.6–3.3)	3.5 (2.9–4.0)	0.13
Forced expiratory volume in 1 second, L	2.4 (2.1–2.8)	2.8 (2.3–3.3)	0.17
Peak expiratory flow, L s^−1^	5.8 (5.0–6.6)	6.1 (5.0–7.2)	0.65
Percent decrease from baseline:			
Forced vital capacity, L	29 (24–34)	24 (17–32)	0.28
Forced expiratory volume in 1 second, L	27 (20–35)	22 (14–31)	0.36
Peak expiratory flow, L s^−1^	24 (17–31)	25 (15–34)	0.88
**2 h after surgery**			
Forced vital capacity, L	2.9 (2.5–3.3)	3.5 (3.0–3.9)	0.06
Forced expiratory volume in 1 s, L	2.4 (2.0–2.8)	2.7 (2.3–3.1)	0.24
Peak expiratory flow, L s^−1^	5.4 (4.6–6.2)	5.9 (5.1–6.7)	0.37
Percent decrease from baseline:			
Forced vital capacity, L	32 (26–37)	26 (21–32)	0.15
Forced expiratory volume in 1 s, L	28 (21–36)	25 (19–32)	0.53
Peak expiratory flow, L.s^−1^	29 (21–37)	27 (20–35)	0.71
**24 h after surgery**			
Forced vital capacity, L	3.7 (3.3–4.0)	3.9 (3.5–4.4)	0.34
Forced expiratory volume in 1 s, L	3.1 (2.8–3.4)	3.2 (2.9–3.5)	0.77
Peak expiratory flow, L s^−1^	7.2 (6.4–8.0)	7.2 (6.1–8.2)	0.92
Percent decrease from baseline:			
Forced vital capacity, L	11 (5.8–15)	15 (10–20)	0.17
Forced expiratory volume in 1 s, L	5.2 (1.4–8.9)	11 (6.2–15)	0.06
Peak expiratory flow, L s^−1^	3.4 (–5.5 to 12)	12 (3.8–19)	0.16

**Fig 3:**
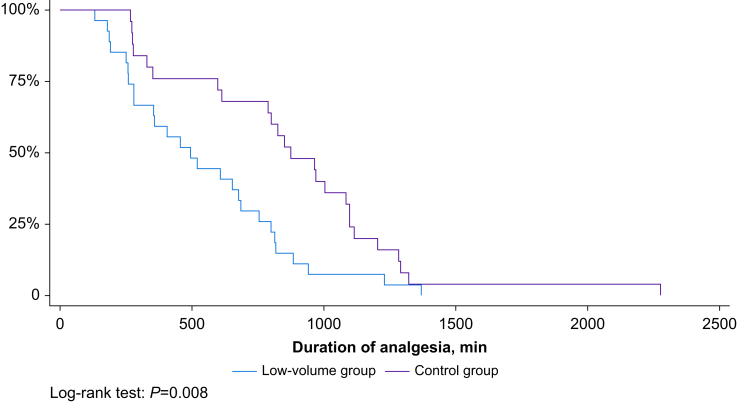
Kaplan–Meier curve showing the duration of analgesia based on the volume of local anaesthetic injected (20 ml [control] *vs* 10 ml [low volume]).

Participants in the low-volume group had longer onset times for sensory and motor blocks, and shorter durations of sensory block, motor block, and analgesia (Fig. 3); they had also consumed significantly more i.v. morphine by 24 h after surgery (Table 3).

**Table 3:** Block-related outcomes. Data are presented as mean and 95% confidence interval or absolute number (proportion) as appropriate. N/A, not applicable; NRS, numeric rating scale.

	Control group (*n*=27)	Low-volume group (*n*=28)	*P*-value
**Onset and duration**			
Onset time of sensory block, min	21 (17–24)	27 (21–33)	0.048
Onset time of motor block, min	20 (17–23)	26 (21–31)	0.037
Duration of sensory block, min	1022 (926–1117)	806 (695–918)	<0.01
Duration of motor block, min	995 (897–1092)	730 (620–841)	<0.001
Duration of analgesia, min	873 (685–1062)	550 (420–679)	<0.01
**2 h after surgery**			
Dyspnoea, *n* (%)	2 (7)	1 (4)	0.61
Claude Bernard-Horner syndrome, *n* (%)	1 (4)	0 (0)	0.49
Hoarseness, *n* (%)	0 (0)	0 (0)	N/A
I.V. morphine consumption, mg	0 (0–1)	2 (0–4)	0.11
Rest pain score (on an NRS from 0–10)	0.4 (0–0.8)	0.8 (0.3–1.3)	0.16
Dynamic pain score (on an NRS from 0–10)	0.4 (0–0.8)	1 (0.4–1.8)	0.07
**24 h after surgery**			
Dyspnoea, *n* (%)	0 (0)	0 (0)	N/A
Claude Bernard-Horner syndrome, n (%)	0 (0)	0 (0)	N/A
Hoarseness, *n* (%)	0 (0)	0 (0)	N/A
I.V. morphine consumption, mg	12 (8–17)	20 (15–25)	0.03
Rest pain score (on an NRS from 0–10)	3.2 (2.3–4.2)	3.3 (2.3–4.3)	0.88
Dynamic pain scores (on an NRS from 0–10)	4.7 (3.8–5.7)	5.3 (4.3–6.3)	0.43
Satisfaction score (on an NRS from 0–10)	8.9 (8.3–9.4)	8.8 (8.1–9.4)	0.80

## Discussion

The results of this double-blinded randomised controlled trial suggest that reducing the volume of local anaesthetic from 20 ml to 10 ml when performing an ISB with an extrafascial approach reduced the rate of hemidiaphragmatic paralysis by 60%, but without any impact on respiratory function. However, reducing the volume of local anaesthetic also reduced the duration of analgesia by more than 5 h and increased i.v. morphine consumption at 24 h after surgery by 8 mg. Both of these differences are clinically relevant. These data generally support the current body of evidence in the literature, suggesting that the probability of hemidiaphragmatic paresis after injection of local anaesthetic near the brachial plexus above the clavicle is relatively high and is nonlinearly related to volume.

Several authors have reported data that explored the potential for reducing hemidiaphragmatic paralysis by reducing the volume injected within the interscalene groove, and these data show conflicting results.^11^, ^12^, ^13^ Although reducing the volume from 20 ml to 5 or 10 ml led to a 55%^11^ and 70%^13^ reduction in hemidiaphragmatic paralysis, respectively, another study did not find any difference in the rate of hemidiaphragmatic paralysis between volumes of 20 and 10 ml.^12^ The main difference between these previous three studies and ours is the method used to measure hemidiaphragmatic paralysis. Two of the studies relied on the paradoxical movement of the diaphragm, and the third defined hemidiaphragmatic paralysis as an inspiratory-to-expiratory hemidiaphragmatic thickness ratio of <1.2, measured at the anterior axillary line bilaterally. In contrast, we chose to assess hemidiaphragmatic excursion with a subcostal approach^8^ because we believe this method to be more sensitive.^2^^,^^3^ Interestingly, our previous study showed a paresis rate of 21% (95% CI 6–46%) with 20 ml bupivacaine 0.5% injected extrafascially,^2^ whereas the rate with 20 ml ropivacaine 0.75% in the current study was 80% (95% CI 61–91%), and the rate with 10 ml ropivacaine in the current study was more comparable with our previous data (19% [95% CI 8–40%]). The difference in paresis rate between the 20 ml volume injection in this study *vs* the previous study might be partly explained by the difference in the type of medication and concentration injected (bupivacaine 0.5% with epinephrine 1:200 000 *vs* ropivacaine 0.75%). Another explanation might be the presence of a deep cervical fascia containing three layers. More specifically, the prevertebral fascial sheath extends from the ligamentum nuchal to the axilla and encompasses the scalene muscles, the brachial plexus, and the phrenic nerve. As a result, any local anaesthetic deposited within this sheath might reach the phrenic nerve through the pressure induced by the medication spread, with subsequent hemidiaphragmatic paresis.

From a clinical perspective, we unfortunately do not have any data that allow us to predict which patients are at risk of developing hemidiaphragmatic paralysis, even if a low-volume extrafascial injection is performed. This uncertainty creates a challenge when managing patients who have grade III or IV dyspnoea secondary to pre-existing respiratory disease or obesity, and who are likely to poorly tolerate hemidiaphragm paralysis. When such patients are undergoing shoulder surgery, the physician should properly assess and discuss the risk–benefit balance of the procedure (risk of respiratory dysfunction *vs* optimal analgesia) with the patient. However, in the absence of ISB, higher opioid requirements could lead to sedation, respiratory depression, and hypoxaemia. We suggest that the ISB technique should be used with caution in patients with advanced lung disease, bearing in mind that a more distal approach such as a combined suprascapular and axillary nerve block technique might be an option.^7^

The shorter duration of analgesia in the low-volume group is not surprising because analgesia duration is directly related to the total mass of local anaesthetic, defined as the volume multiplied by the concentration of the medication. In other words, the higher the volume or the higher the concentration used, the longer the duration of the block will be. Indeed, Fredrickson and colleagues^14^ have reported that increasing the volume of ropivacaine 0.375% from 10 ml to 40 ml for an interscalene block in 185 patients undergoing shoulder surgery increased the median duration of the sensory block by 5 h, and that increasing the concentration of a 20 ml injection volume from 0.375% to 0.75% will prolong the sensory block by a median of 3 h. Historically, volumes of up to 30 ml^15^ or 40 ml^16^ were injected in the interscalene region with the nerve-stimulator technique. In contemporary clinical practice, a volume of 20 ml seems to be a reasonable compromise between a satisfactory clinical effect and safety, without causing too much discomfort. We therefore advocate injecting a volume of 20 ml for an ISB with an extrafascial approach, although some physicians might have a different opinion. Furthermore, a low volume is associated with a longer time to the onset of sensory and motor blocks (by 6 min). The clinical relevance of this increase is disputable and depends on the environment and the setting of the operating theatre.

This study has several limitations. Firstly, we acknowledge that the fascia cannot be accurately identified using our ultrasound machine, and therefore describing our approach as ‘extrafascial’ might be somewhat inaccurate. However, as previously mentioned, ‘the concept of an extrafascial injection can be easily translated by respecting a short distance between the needle tip and the lateral border of the brachial plexus, while ensuring that the local anaesthetic injectate spreads towards the nerve roots’.^2^ In addition, as reported in the methods section, we did not reposition the needle and elected to perform an intention-to-treat analysis to better reflect daily clinical practice. Secondly, we did not evaluate the impact of the intramuscular injection of local anaesthetic within the middle scalene muscle. While myotoxicity after injection of bupivacaine into extraocular muscles has been well documented,^17^^,^^18^ clinical experience before the era of ultrasound indicated that unintentional intramuscular injection of local anaesthetic was not problematic. Thirdly, supplemental oxygen requirement could have been an outcome of interest to further measure the clinical impact of the block and should probably be considered for future studies in this field. Furthermore, having grade III or IV dyspnoea as an exclusion criterion means that we cannot ignore the potential for hemidiaphragmatic paresis to have a more important impact on respiratory function in the presence of respiratory comorbidities. Additional studies are needed to investigate this further. It should also be noted that the patients in our study underwent surgery during general anaesthesia, and therefore our findings need to be confirmed in individuals undergoing awake shoulder surgery because ambulatory shoulder arthroscopy is performed under regional anaesthesia only in many centres. Finally, the conclusions of this study cannot be translated to other types of peripheral nerve block. Further studies investigating the pharmacodynamic properties of the volume of extrafascially injected local anaesthetic are needed to define the optimal anaesthetic volume.

In conclusion, a low volume of local anaesthetic injected extrafascially reduces the rate of hemidiaphragmatic paralysis without impacting on the respiratory function when compared with a higher volume, but at the expense of increased time to onset of action and reduced postoperative analgesia. While balancing risks and benefits, injecting 20 ml of anaesthetic extrafascially for an interscalene block, or lateral to the outer border of the brachial plexus, seems to be a reasonable approach.

## Authors' contributions

Patient recruitment and data collection: YR

Manuscript editing: SG

Patient recruitment and data collection: EG

Statistical analysis: JRB

Manuscript editing: PG

Study design, data interpretation, and primary manuscript preparation: EA

## Declaration of interest

EA received grants from the Swiss Academy for Anesthesia Research (SACAR) and the Swiss National Science Foundation, Bern, Switzerland to support his clinical research. All other authors declare no competing interests.
